# An assessment of the environmental and socio-economic impacts of alien rabbits and hares

**DOI:** 10.1007/s13280-021-01642-7

**Published:** 2021-10-28

**Authors:** Tom Allmert, Jonathan M. Jeschke, Thomas Evans

**Affiliations:** 1 Institute of Biology, Freie Universität Berlin, Königin-Luise-Str. 1-3, 14195 Berlin, Germany; 2 Leibniz Institute of Freshwater Ecology and Inland Fisheries (IGB), Müggelseedamm 310, 12587 Berlin, Germany; 3 Berlin-Brandenburg Institute of Advanced Biodiversity Research (BBIB), Königin-Luise-Str. 2-4, 14195 Berlin, Germany; 4 Present Address: Department of Biology, Humboldt-Universität zu Berlin, Invalidenstr. 42, 10115 Berlin, Germany

**Keywords:** European rabbit, Extinction, Grazing, Human well-being, Invasive alien species, Leporid

## Abstract

Directly comparable data on the environmental and socio-economic impacts of alien species informs the effective prioritisation of their management. We used two frameworks, the Environmental Impact Classification for Alien Taxa (EICAT) and Socio-Economic Impact Classification for Alien Taxa (SEICAT), to create a unified dataset on the severity and type of impacts caused by alien leporids (rabbits and hares). Literature was reviewed to collate impact data, which was categorised following EICAT and SEICAT guidelines. We aimed to use these data to identify: (1) alien leporid species with severe impacts, (2) their impact mechanisms, (3) the native species and local communities vulnerable to impacts and (4) knowledge gaps. Native species from a range of taxonomic groups were affected by environmental impacts which tended to be more damaging than socio-economic impacts. Indirect environmental impacts were particularly damaging and underreported. No impact data were found for several alien leporid species.

**Supplementary Information:**

The online version contains supplementary material available at 10.1007/s13280-021-01642-7.

## Introduction

A species that has been deliberately or accidentally introduced by human actions to regions outside of its natural distribution is termed an alien species. If it has adverse impacts on native biodiversity, economic development or human well-being, it is termed an invasive alien species (IUCN 2021a). Examples of such impacts include declines in native vertebrate species in Australia due to predation by the feral cat (*Felis catus*) (Hamer et al. 2021), damage to agriculture in the Philippines caused by the Golden apple snail (*Pomacea canaliculata*) (Naylor 1996) and an outbreak of chikungunya fever on Reunion Island caused by the Tiger mosquito (*Aedes albopictus*) (Josseran et al. 2006).

Across the seven continents of the world, numbers of new alien species are predicted to rise on average by 36% between 2005 and 2050 (Seebens et al. 2021). However, not all new alien species are destined to have severe impacts. There are approximately 12 000 known alien species in Europe, of which about 15% are invasive (European Commission 2013). Given their vast number, identifying and managing the impacts of all alien species is an unrealistic expectation. To effectively protect biodiversity and human well-being, a pragmatic approach is required that prioritises the most damaging alien species, as well as the most vulnerable native species and human populations (Pyšek et al. 2020). Indeed, prioritisation was a central requirement of Aichi Target 9 of the Convention on Biodiversity (CBD), which by 2020 sought to identify and prioritise invasive alien species and their pathways, and to control and eradicate priority species (CBD 2021a). Prioritisation will also be a requirement of the post-2020 targets (CBD 2021b), and the EU Biodiversity Strategy for 2030 prioritises the protection of native species that are known to be threatened by alien species (European Commission 2020).

However, unified, directly comparable data on the impacts of alien species is often unavailable (Blackburn et al. 2014; Jeschke et al. 2014; Kumschick et al. 2015; Nentwig et al. 2016). This makes it difficult to meaningfully compare the severity of impacts caused by different alien species and sustained by different native species and human populations. Yet these comparisons are required in order to prioritise the most damaging alien species and the most vulnerable native species and human populations.

In recognition of this problem, two protocols have been developed to categorise the severity and type of environmental and socio-economic impacts caused by alien species: the Environmental Impact Classification for Alien Taxa (EICAT; Blackburn et al. 2014; Kumschick et al. 2020) and the Socio-Economic Impact Classification for Alien Taxa (SEICAT; Bacher et al. 2018). In much the same way that the IUCN Red List of Threatened Species (www.iucnredlist.org) was developed to provide evidence to inform decisions regarding the conservation of threatened species (IUCN 2021b), EICAT and SEICAT have been developed to provide evidence to inform decisions regarding the management of alien species.

EICAT is a simple, objective and transparent method that may be used to identify differences in the severity and type of impacts caused by alien species, facilitating clear comparisons of these impacts. It may enable a better understanding of the severity and type of impacts caused by different alien taxa, alert stakeholders to the possible consequences of the introduction of a specific alien species, and inform management actions to mitigate impacts (IUCN 2020a). EICAT was recently adopted by the IUCN and includes published criteria (IUCN 2020a) and guidelines (IUCN 2020b) to inform the assessment process. It has so far been applied to alien species from several taxonomic groups including birds (Evans et al. 2016), amphibians (Kumschick et al. 2017), fish (Galanidi et al. 2018), gastropods (Kesner and Kumschick 2018) and bamboos (Canavan et al. 2019). The data produced have been used to identify factors associated with alien species that influence the severity of their impacts (Evans et al. 2018a) and characteristics of native species that increase their vulnerability to the impacts of alien species (Evans et al. 2021). EICAT assessments may therefore assist in prioritising management interventions towards damaging alien species and vulnerable native species. EICAT assessments have also been used to identify the factors that cause impact data to be available for some alien species (Evans et al. 2018b) and some regions of the world (Evans and Blackburn 2020), but not others. EICAT assessments may therefore also be used to prioritise research towards data-deficient alien species and regions (of which there are many, e.g. Evans 2021). SEICAT has so far been applied to categorise the socio-economic impacts of alien amphibians (Bacher et al. 2018), gastropods (Kesner and Kumschick 2018), fish (Galanidi et al. 2018) and birds (Evans et al. 2020). These assessments identified damaging impacts on economic development and human well-being in communities around the world. They also revealed that for many regions, data on these impacts is scarce, and the results may therefore be used to prioritise future research to identify impacts. SEICAT includes published criteria to guide the assessment process (Bacher et al. 2018).

Of the 62 species from the Leporidae family (hares and rabbits) (Ge et al. 2013; Wilson et al. 2016), 12 have been introduced to new locations around the world (Bssarbar and Lambertucci 2018). For example, the European hare (*Lepus europaeus*), which originates from Europe and Eastern Asia, has been introduced to locations including North and South America, Oceania and other parts of Europe. The European rabbit (*Oryctolagus cuniculus*), which originates from the Iberian Peninsula, has been introduced to South America, Oceania, other parts of Europe and many islands (Bssarbar and Lambertucci 2018). Alien hares and rabbits (hereafter alien leporids) can have damaging environmental impacts. For example, in Italy, introduced Eastern cottontails (*Sylvilagus floridanus*) are preyed on by red foxes (*Vulpes vulpes*), which increases the red fox population size, and in-turn, red fox predation of native European hares (Cerri et al. 2017). Alien leporids can also have damaging socio-economic impacts. For example, in Australia in 2004, competition with sheep for grazing by introduced European rabbits may have cost the wool production industry AU$ 32.38 million (Vere et al. 2004).

Although many studies have examined the environmental and socio-economic impacts of specific alien leporid species, none have categorised and assessed their impacts using scoring systems, and this remains a significant research gap for invasion science. The objective of this study was to undertake the first global EICAT and SEICAT assessments for all known alien leporid species, and in so doing, create the first directly comparable dataset on the severity and type of their environmental and socio-economic impacts. We aimed to use these data to: (1) identify the alien leporid species with the most damaging environmental and socio-economic impacts; (2) identify the environmental and socio-economic impact mechanisms associated with alien leporid species, including those that are particularly damaging; (3) identify the regions, native species and local communities that are particularly vulnerable to the environmental and socio-economic impacts of alien leporid species; and (4) identify knowledge gaps regarding the severity, type and geographic distribution of impacts caused by alien leporid species. In so doing, we aimed to provide information that may inform management actions for alien leporids, and to direct future research to regions where knowledge on their impacts is lacking.

## Materials and methods

### Data

We undertook a literature review (see Supplementary Information, Appendix S1 for details) to identify data describing the environmental and socio-economic impacts of ten hare and two rabbit species with known alien populations worldwide (as identified in a recent global review of the distribution of alien leporid species) (Barbar and Lambertucci 2018). These species are the Arctic hare (*Lepus arcticus*), Black-tailed jackrabbit (*L. californicus*), Cape hare (*L. capensis*), Corsican hare (*L. corsicanus*), Eastern cottontail (*Sylvilagus floridanus*), European hare (*L. europaeus*), European rabbit (*O. cuniculus*), Iberian hare (*L. granatensis*), Indian hare (*L. nigricollis*), Mountain hare (*L. timidus*), Snowshoe hare (*L. americanus*) and White-tailed jackrabbit (*L. townsendii*).

Following Evans et al. (2016), we carried out an online search using search terms within a search string, in conjunction with the specific alien species’ scientific and common name(s). For example, the search string for the Snowshoe hare was: (“introduced species”, “invasive species”, “invasive alien species”, “IAS”, “alien”, “non-native”, “non-indigenous”, “pest”, “feral” and “exotic”) AND (“snowshoe hare” OR “snowshoe rabbit” OR “varying hare” OR “*Lepus americanus*”). We searched the Web of Science (http://apps.webofknowledge.com), Google (https://www.google.co.uk) and Google Scholar (https://scholar.google.co.uk). We also reviewed the IUCN Red List of Threatened Species (http://www.iucnredlist.org), the CABI Invasive Species Compendium (http://www.cabi.org/isc) and the Global Invasive Species Database (GISD) of the Invasive Species Specialist Group (ISSG) (http://www.iucngisd.org). We searched for additional references listed in any articles and data sources found, repeating this process to a point where no new sources of data were identified. We selected publications based on the information provided in the titles and abstracts.

Our searches were carried out in English, and therefore we may have missed impact reports written in other languages. However, alien leporids are distributed across Western Europe, Australasia, North and South America, and many islands. Most of these regions (aside from South America) are broadly English speaking and/or publish scientific research in English (including many islands such as Saint Helena, Ascension Island, Seychelles, Kiribati, Mauritius, Hawaii and the Northwestern Hawaiian Islands). Furthermore, many non-English speaking regions (particularly in Western Europe) publish their news in both their native language and in English.

#### Environmental impacts

**Fig. 1:**
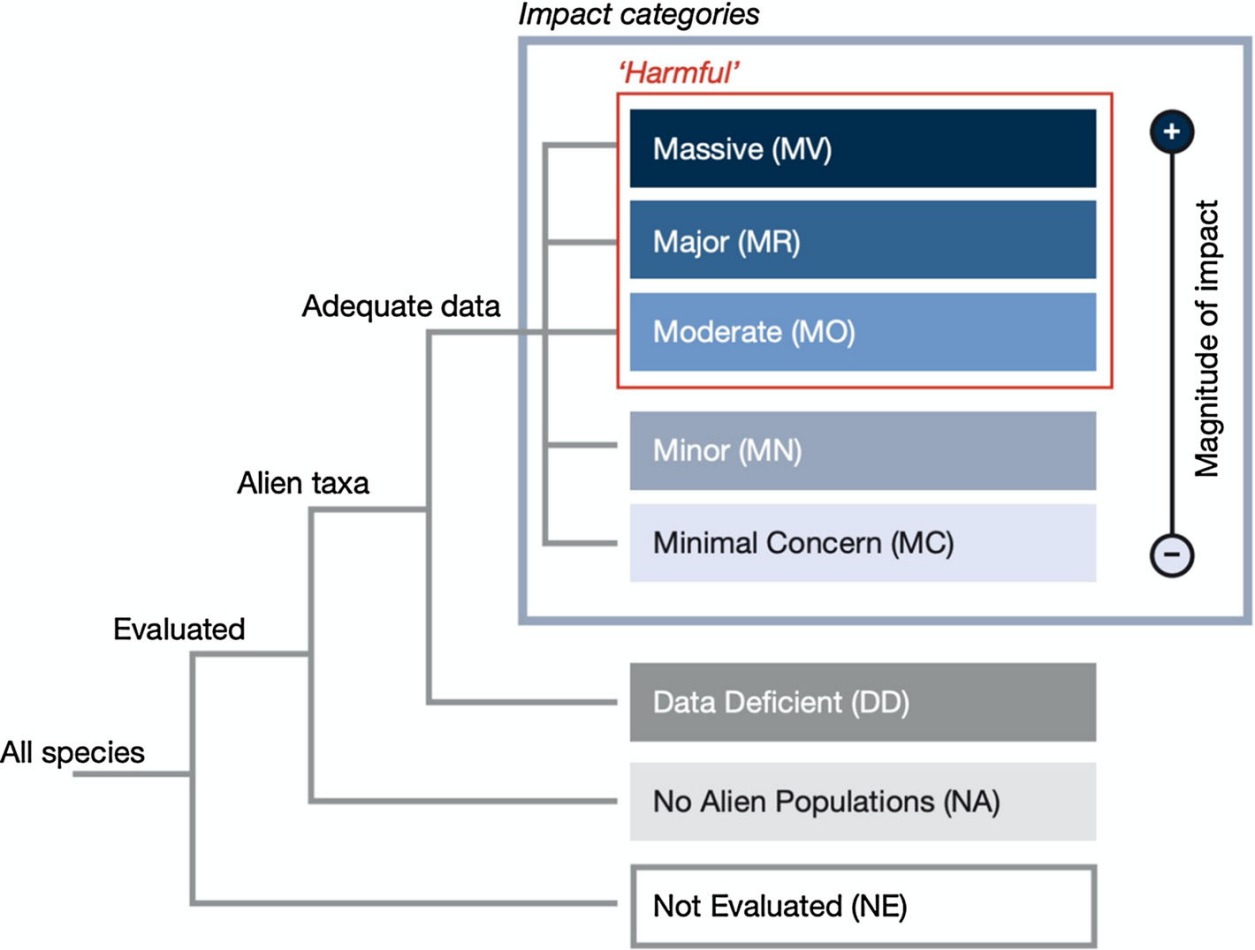
The criteria used under EICAT and SEICAT to categorise the severity of environmental and socio-economic impacts of alien leporid species Reproduced from IUCN (2020a) after Blackburn et al. (2014) and Bacher et al. (2018)

For each data source found, we allocated each alien leporid species and every native species it affected into one of five impact categories (Fig. 1), depending on the severity of the documented environmental impacts: Minimal Concern (MC—whilst the alien species interacted with a native species, it caused no discernible impacts), Minor (MN—the alien species caused impacts that affected the performance of individual native species), Moderate (MO—the alien species caused declining populations of one or more native species), Major (MR—the alien species caused native species extirpations that would be reversible if the alien species was removed), Massive (MV—the alien species caused irreversible native species extinctions). We also categorised each impact by its type using 12 EICAT impact mechanisms: (1) Competition, (2) Predation, (3) Hybridisation, (4) Transmission of disease, (5) Parasitism, (6) Poisoning/toxicity, (7) Bio-fouling or other direct physical disturbance, (8) Grazing/herbivory/browsing (hereafter ‘Grazing’), (9, 10, 11) Chemical, physical, or structural impact on ecosystem and (12) Indirect impacts through interactions with other species (hereafter ‘Indirect impacts’).

When allocating each alien leporid species and affected native species to the appropriate impact category (MC–MV), we followed the criteria in Table 1 of the IUCN EICAT Categories and Criteria (IUCN 2020a), the revisions to these criteria (Volery et al. 2020) and the EICAT guidelines (IUCN 2020b). For example, the Indian hare grazes on *Stenotaphrum dimidiatum*, a native grass (*Poaceae*) species on Cousin Island (Seychelles). However, there is no evidence to indicate that this has caused a decline in the distribution of this grass species on the island (Kirk and Racey 1992). As such, the recorded impacts match the criteria for the MN impact category and the ‘grazing’ impact mechanism. We counted an impact between an alien leporid species and a specified native species as being one impact record. Therefore, for example, a data source describing impacts by an alien leporid species on five different native species would result in five separate impact records.

**Table 1:** Assessment summary table showing the number, type and severity of impact records for each alien leporid species

Scoring protocol	Impact mechanism	Impact severity	Arctic hare	Black-tailed jackrabbit	Cape hare	Corsican hare	Eastern cottontail	European hare	European rabbit	Iberian hare	Indian hare	Mountain hare	Snowshoe hare	White-tailed jackrabbit	Total
EICAT	Competition	Weak				9		2	1						12
		Moderate					1	2	2						5
		Severe							1						1
	Grazing	Weak						4	5		7	6			22
		Moderate						1	50				9		60
		Severe							5						5
	Hybridisation	Weak													
		Moderate						3							3
		Severe													
	Disease	Weak								1					1
		Moderate													
		Severe													
	Indirect	Weak							3						3
		Moderate					1		30						31
		Severe							8						8
SEICAT	Agriculture	Weak					2	7	19		1				29
		Moderate													
		Severe							3						3
	Health	Weak		1					2						3
		Moderate													
		Severe													
	Material	Weak							2						2
		Moderate													
		Severe													
	Recreation	Weak					1		2						3
		Moderate										1			1
		Severe													
	Tourism	Weak													
		Moderate							1						1
		Severe													
	Total			1		9	5	19	134	1	8	7	9		193

#### Socio-economic impacts

SEICAT uses changes in human activities caused by an alien species as a common metric for assessing impacts to human well-being (Bacher et al. 2018). It adopts the same five impact categories as EICAT (MC–MV). Based on the literature found, we allocated each alien leporid species to one of these categories, depending on its most severe impact to human well-being: MC—the alien species did not affect human well-being; MN—the alien species made it difficult for people to participate in their normal activities, and individuals suffered in at least one constituent of human well-being (e.g. security, material assets, health); MO—the alien species caused a reduction in the size of an activity, with fewer people participating in it; MR—the alien species caused the local disappearance of an activity from all or part of an area invaded by an alien taxon, but this impact would most likely be reversible if the alien species was controlled or removed; MV—the alien species caused the local disappearance of an activity and this change would most likely persist for at least a decade, even if the alien species was controlled or removed.

Unlike EICAT, SEICAT does not include a standardised set of impact mechanisms to be used to categorise alien species by the types of impact that they have. This is because such impacts may vary widely, depending on the types of communities affected. Instead, these mechanisms are determined during the SEICAT assessment by the assessor. For this assessment, we identified five broad mechanisms through which alien leporids may affect human well-being. These are impacts on: (1) agriculture/forestry/horticulture industries (hereafter ‘agriculture’), (2) the tourism industry, (3) human health and safety, (4) recreation, and (5) material assets. For example, on Macquarie Island, grazing by the European rabbit has destabilised coastal slopes, preventing tourists from using boardwalks to view wildlife, and this has reduced the visitor numbers (Tasmania National Parks Association 2006). As such, recorded impacts match the MO impact category and the tourism impact mechanism. Under both EICAT and SECIAT, if no information on the impacts of an alien species is available, it is classified as being Data Deficient (DD).

To minimise the potential for subjectivity to influence the assessment, scoring of impacts was undertaken by T.A. and reviewed by T.E.; any uncertainties were discussed by all three authors. The EICAT and SEICAT criteria used to guide the assessment process have been developed with the aim of minimising subjectivity, and EICAT and SEICAT assessments have been successfully completed and published using these criteria (e.g. Canavan et al. 2019; Evans et al. 2020).

### Analysis

All analyses were carried out in R version 4.0.0 (R Core Team 2020). We compared the number of environmental and socio-economic impact records associated with alien leporid species using Pearson’s product–moment correlation (Best and Roberts 1975). We used contingency table tests (unconditional exact tests: the FunChisq package Zhong and Song 2019) to analyse the actual and expected distribution of environmental and socio-economic impact records across alien leporid species that caused impacts, the native species that they affected, impact severity, impact type (mechanism) and impact location. Each contingency table result includes an ‘estimate’ (produced by the FunChisq package) which is a value between 0 and 1, where 1 indicates complete mathematical dependence of the two variables, and 0 indicates complete independence.

We analysed impacts at the continental scale using the following regions: Asia, Oceania, Europe, North America, South America and islands. Following Evans et al. (2020), due to the relatively small size of our impact dataset, impact severity data were converted into a three-level response variable: ‘weak’ impacts = MC or MN under EICAT or SEICAT; ‘moderate’ = MO; and ‘severe’ = MR or MV. For the contingency table tests, due to small sample sizes amongst some categories of interest, we grouped some of these categories. These groups are described with the contingency tables (Supplementary Information, Appendix S2).

## Results

### Environmental vs socio-economic impacts

**Fig. 2:**
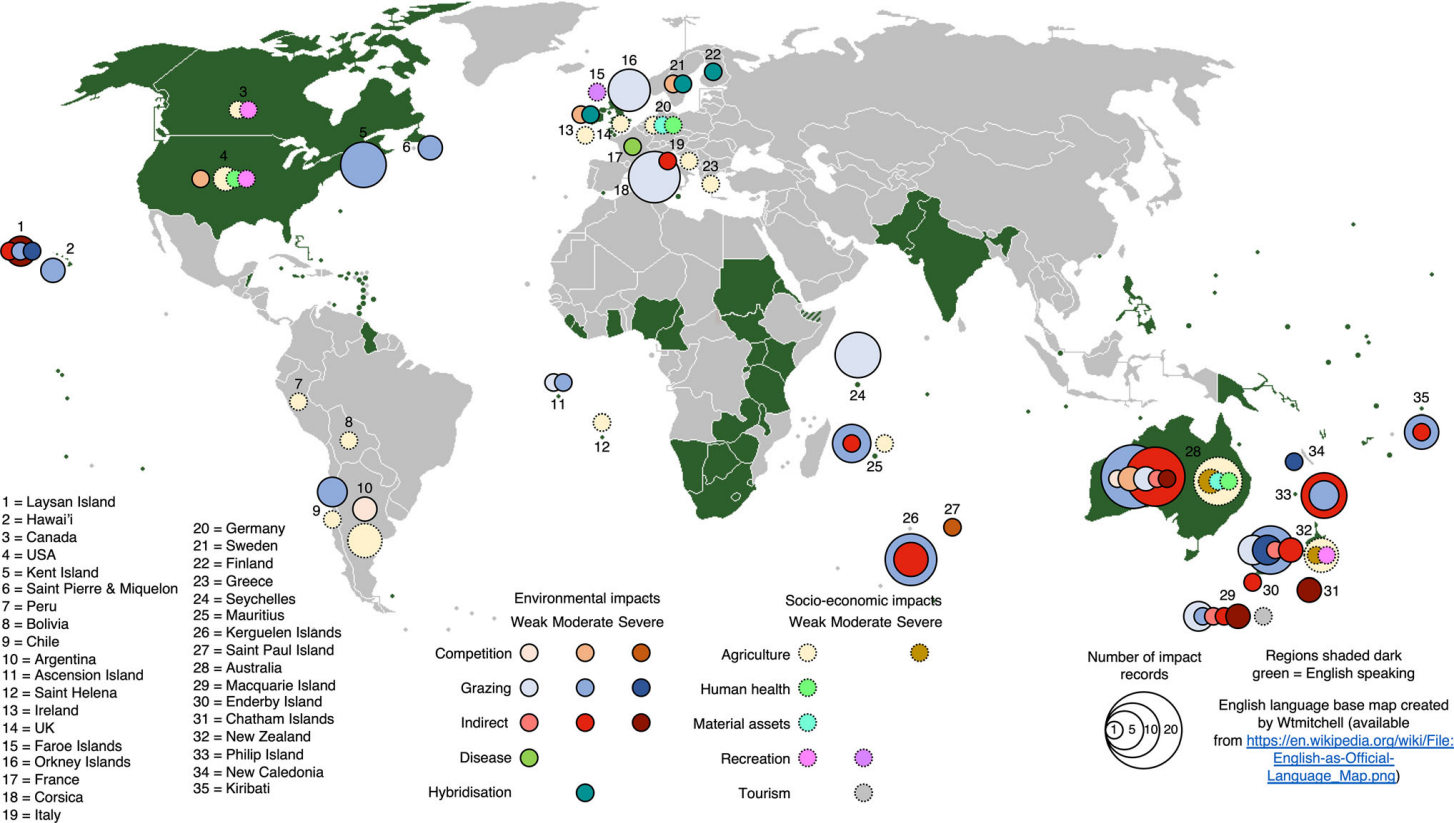
The global distribution of the environmental and socio-economic impacts of alien leporid species as categorised using EICAT and SEICAT

**Fig. 3:**
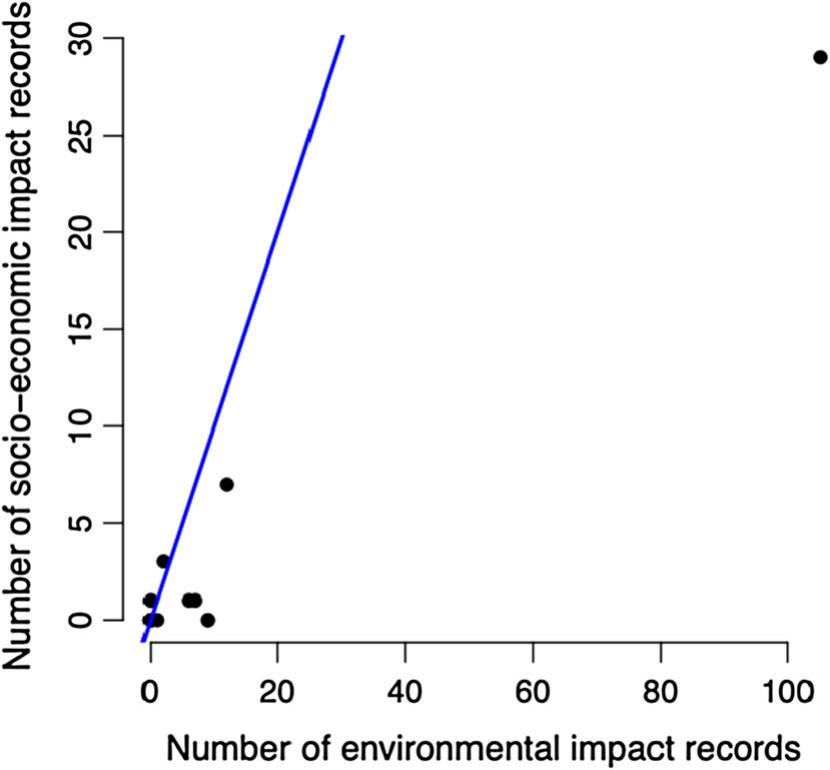
The correlation between the number of environmental impact records and the number of socio-economic impacts records found for alien leporid species (Pearson’s product–moment correlation: *r* = 0.98, df = 10, *P* < 0.001)

The literature review identified many more environmental than socio-economic impact records (151 and 42, respectively; Table 1; Fig. 2). The number of environmental and socio-economic impacts caused by alien leporid species was positively correlated (*r* = 0.98, df = 10, *P* < 0.001, Table 2; Fig. 3) and nonrandomly distributed: the Eastern cottontail caused more socio-economic impacts than would be expected by chance (Table 3, test #1) (Table 3 provides a summary of all contingency table test results; the complete contingency table tests are provided in Supplementary Information, Appendix S2).

**Table 2:** The number of environmental and socio-economic impact records for each alien leporid species

	Environmental impact records	Socio-economic impact records	Total impact records
European rabbit	105	29	134
European hare	12	7	19
Corsican hare	9	0	9
Snowshoe hare	9	0	9
Indian hare	7	1	8
Mountain hare	6	1	7
Eastern cottontail	2	3	5
Iberian hare	1	0	1
Black-tailed jackrabbit	0	1	1
White-tailed jackrabbit	0	0	0
Cape hare	0	0	0
Arctic hare	0	0	0

**Table 3:** Contingency table test results summary (see Supplementary Information, Appendix S2 for full contingency table test results)

Data tested	Test number and description	Result description	*χ*^2^	df	*P*	Est	Full test result ref. (Supplementary Information, Appendix S2)	Related figure references
Environmental and socio-economic impact records	#1. The association between the number of ‘weak’, ‘moderate’ and ‘severe’ environmental and socio-economic impact records as distributed by alien leporid species	Impact severity was nonrandomly distributed: in particular, the Eastern cottontail caused more socio-economic impacts than expected	5.04	7	< 0.001	0.08	Table S1	NA
	#2. The association between the number of ‘weak’, ‘moderate’ and ‘severe’ environmental and socio-economic impact records	Impact severity was nonrandomly distributed: there were more ‘weak’ and fewer ‘moderate’ socio-economic impacts than expected, and fewer ‘weak’ and more ‘moderate’ environmental impacts than expected	38.73	2	< 0.001	0.44	Table S2	NA
	#3. The association between the number of environmental and socio-economic impact records as distributed by broad geographic location	Impacts were nonrandomly distributed: in particular, there were fewer socio-economic impacts on islands, and more in North and South America than expected	48.97	4	< 0.001	0.32	Table S3	NA
Environmental impact records	#4. The association between the number of ‘weak’, ‘moderate’ and ‘severe’ impact records as distributed by alien leporid species	Impact severity was nonrandomly distributed: in particular, the Corsican hare, Indian hare and Mountain hare caused more ‘weak’ impacts than expected, and the European rabbit caused fewer ‘weak’ impacts than expected	104.67	12	< 0.001	0.39	Table S4	Figure 4A
	#5. The association between the number of ‘weak’, ‘moderate’ and ‘severe’ impact records as distributed by family of affected native plants	Not significant	3.97	2	0.063	0.2	Table S5	Figure S1 (Supplementary Information, Appendix S3)
	#6. The association between the number of ‘weak’, ‘moderate’ and ‘severe’ impact records as distributed by class of affected native animals	Not significant	8.68	4	0.059	0.29	Table S6	Figure 4B
	#7. The association between the number of ‘weak’, ‘moderate’ and ‘severe’ impact records as distributed by animal and plant kingdom	Impact severity was nonrandomly distributed: impacts on native animals tended to be more severe than expected	9.04	2	0.007	0.24	Table S7	NA
	#8. The association between the number of ‘weak’, ‘moderate’ and ‘severe’ impact records as distributed by broad geographic location	Not significant	9.06	6	0.096	0.15	Table S8	Figure 4C
	#9. The association between the number of ‘weak’, ‘moderate’ and ‘severe’ impact records as distributed by EICAT impact mechanism	Impact severity was nonrandomly distributed: indirect impacts tended to be more severe than expected	21.1	6	0.002	0.23	Table S9	Figure 4D
	#10. The association between the number of impact records for each alien leporid species as distributed by EICAT impact mechanism	Impact mechanisms were nonrandomly distributed: in particular, the European hare caused more competition impacts and more impacts through ‘other mechanisms’ (transmission of disease and hybridisation) than expected, and ‘other species’ caused more impacts through ‘other mechanisms’ than expected	76.2	18	< 0.001	0.31	Table S10	Figure 4E
	#11. The association between the number of impact records for each native animal class as distributed by EICAT impact mechanism	Impact mechanisms were nonrandomly distributed: in particular, mammal species sustained more competition impacts than expected	25.28	4	< 0.001	0.49	Table S11	Figure 4F
	#12. The association between the number of impact records for each EICAT impact mechanism as distributed by broad geographic location	Impact mechanisms were nonrandomly distributed: in particular, there were more impacts through ‘other mechanisms’ (transmission of disease and hybridisation) in Europe than expected, and more competition impacts in the Americas than expected	38.33	9	< 0.001	0.29	Table S12	Figure 4G
Socio-economic impact records	#13. The association between the number of ‘weak’ and the number of ‘moderate’ or ‘severe’ impacts as distributed by alien leporid species	Not significant	1.39	3	0.624	0.13	Table S13	Figure 5A
	#14. The association between the number of ‘weak’ and the number of ‘moderate’ or ‘severe’ impacts as distributed by broad geographic location	Not significant	5.63	4	0.176	0.23	Table S14	Figure 5B
	#15. The association between the number of ‘weak’ and the number of ‘moderate’ or ‘severe’ impacts as distributed by SEICAT impact mechanism	Not significant	0.6	1	0.577	0.12	Table S15	Figure 5C
	#16. The association between the number of impact records for each alien leporid species as distributed by SEICAT impact mechanism	Not significant	2.37	3	0.298	0.17	Table S16	Figure 5D
	#17. The association between the number of impact records for each SEICAT impact mechanism as distributed by broad geographic location	Not significant	5.32	4	0.23	0.23	Table S17	Figure 5E

*χ*^*2*^ Chi-square value, *df* degrees of freedom, *P P* value, *Est* estimate (a value between 0 and 1, where 1 indicates complete mathematical dependence of the two variables, and 0 indicates complete independence)

Impact severity was nonrandomly distributed across environmental and socio-economic impacts: there were more ‘weak’ and fewer ‘moderate’ socio-economic impacts, and fewer ‘weak’ and more ‘moderate’ environmental impacts than expected (Table 3, test #2). Environmental and socio-economic impacts were nonrandomly distributed across geographic location: there were fewer socio-economic impacts on islands, and more in North and South America than expected (Table 3, test #3).

### Environmental impacts

**Fig. 4:**
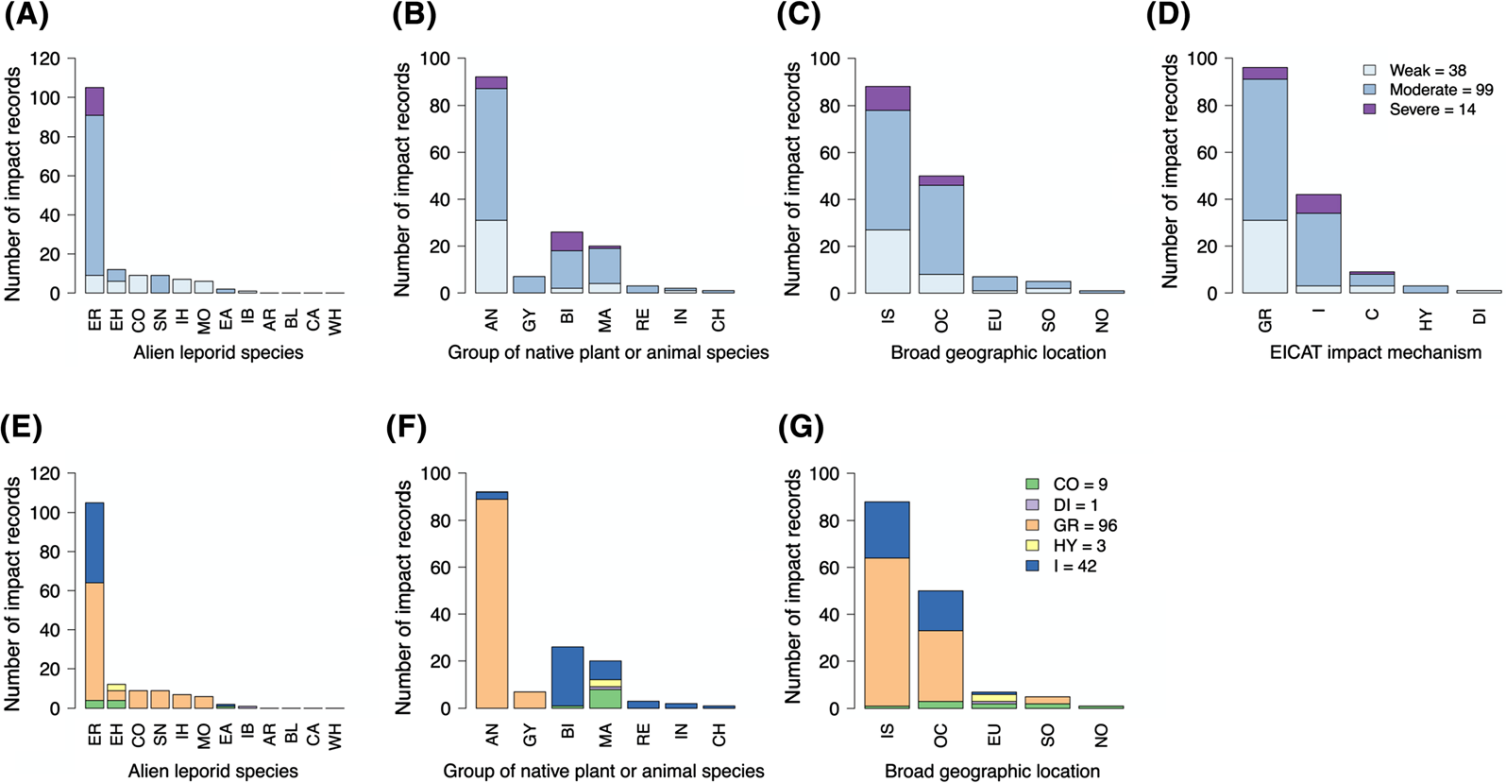
The number and severity of environmental impact records as categorised by: **A** alien species causing impacts, **B** class of native species sustaining impacts, **C** broad geographic location of impact, **D** mechanism of impact; and the number and mechanism of environmental impact records as categorised by: **E** alien species causing impacts, **F** class of native species sustaining impacts, and **G** broad geographic location of impact. For two impact records, the affected organisms were only identified to their taxonomic class (flowering plant, Angiosperms). For all other impact records, affected organisms were identified to species level. Alien leporid species: *AR* Arctic hare, *BL* Black-tailed jackrabbit, *CA *Cape hare, *CO* Corsican hare, *EA* Eastern cottontail, *EH* European hare, *ER* European rabbit, *IB* Iberian hare, *IH* Indian hare, *MO* Mountain hare, *SN* Snowshoe hare, *WH* White-tailed jackrabbit. Native plant classes: *AN* angiosperms, *GY* gymnosperms, *UP* unidentified plant species. Native animal classes: *MA* mammals, *BI* birds, *RE* reptiles, *CH* chilopods, *IN* insects. Locations: *OC* Oceania, *EU* Europe, *IS* Island, *SO* South America, *NO* North America. Impact severity categories: *weak* impacts categorised as MC or MN under EICAT, *moderate* impacts categorised as MO, *severe* impacts categorised as MR or MV. EICAT impact mechanisms: *C* competition, *DI* transmission of diseases to native species, *GR* grazing/herbivory/browsing, *HY* hybridization, *I* indirect impacts through interaction with other species

No impact data were found for four alien leporid species, which were classified as DD under EICAT (Fig. 4A). Most impact records (66%) were ‘moderate’, 25% were ‘weak’ and 9% were ‘severe’. The only species to cause ‘severe’ impacts was the European rabbit. Impact severity was nonrandomly distributed across alien leporid species: several species had more ‘weak’ and fewer ‘moderate’ impacts, and the European rabbit had fewer ‘weak’ and more ‘moderate’ and ‘severe’ impacts than expected (Table 3, test #4).

Most impact records were for plants (66%, Fig. 4B). There was no significant variation in impact severity across plant families (Table 3, test #5). Alien leporids have caused the extirpation or extinction of plant species from several families (Supplementary Information, Appendix S3).

Most impact records on animals were for birds (50%, Fig. 4B). Although there was no significant variation in impact severity across taxonomic class of animal species (Table 3, test #6), eight of the nine ‘severe’ impacts were on birds (Fig. 4B). Impact severity was nonrandomly distributed across animal and plant kingdoms, with impacts on animals being more severe than expected (Table 3, test #7).

Impacts were recorded at five broad geographic locations. Most, including the only ‘severe’ impacts, occurred on islands (58%) or either on mainland Australia or New Zealand (33% combined) (Figs. 2, 4C). There was no significant variation in impact severity across geographic location (Table 3, test #8).

Environmental impacts occurred through five mechanisms (Fig. 4D). Most were caused by grazing (64%) and indirect impacts (28%). Many indirect impacts (67%) were a consequence of grazing by alien leporids. As such, 82% of impacts directly or indirectly resulted from grazing.

Environmental impact mechanisms were nonrandomly distributed across (i) impact severity, (ii) alien leporid species, (iii) class of affected animals and (iv) geographic location. Specifically, there were (i) fewer ‘weak’ and more ‘severe’ indirect impacts than expected (Table 3, test #9), (ii) more impacts through competition and ‘other mechanisms’ (transmission of disease and hybridisation) caused by the European hare than expected (Table 3, test #10; Fig. 4E), (iii) more competition impacts sustained by mammals than expected (Table 3, test #11; Fig. 4F) and (iv) more impacts caused by ‘other mechanisms’ (transmission of disease and hybridisation) in Europe than expected (Table 3, test #12; Fig. 4G).

### Socio-economic impacts

**Fig. 5:**
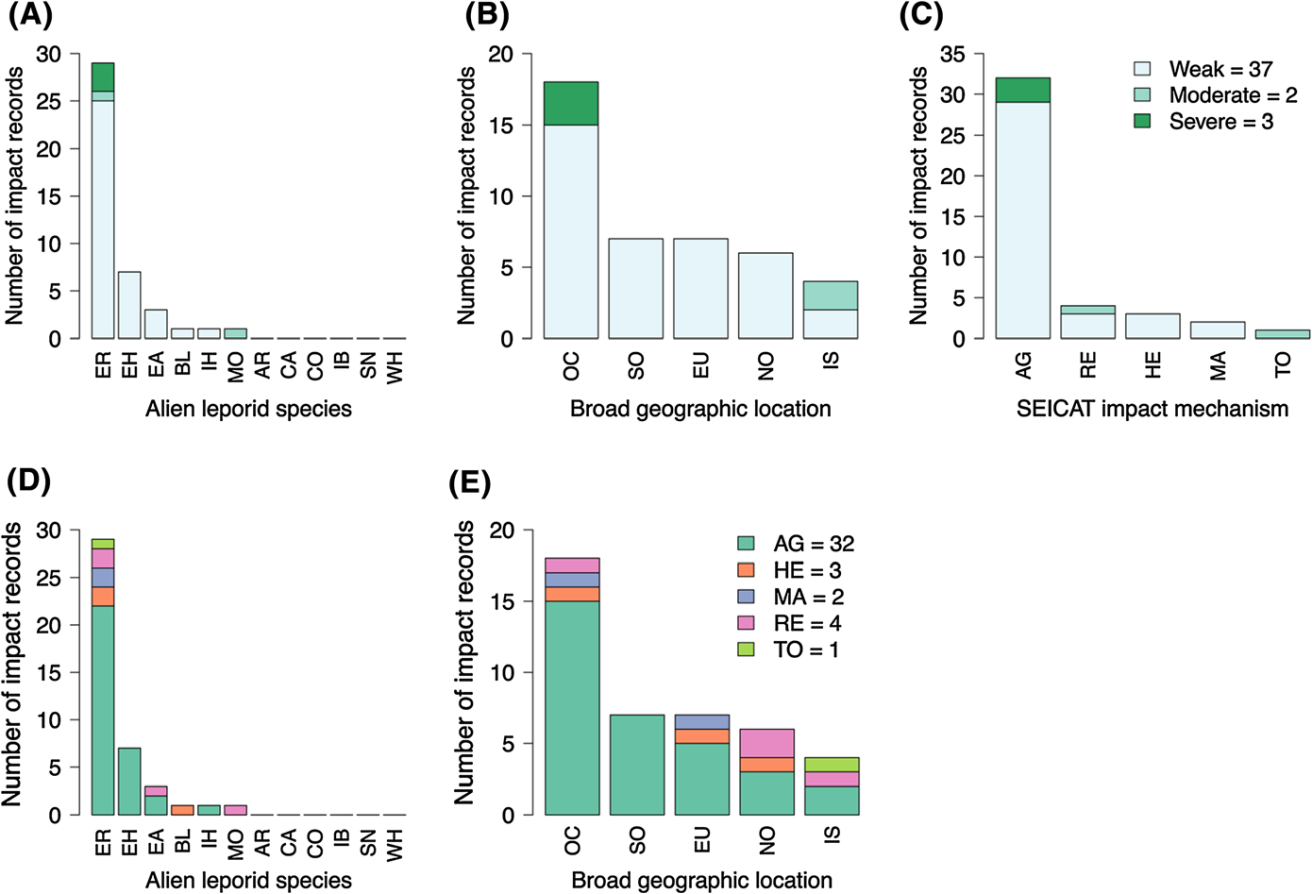
The number and severity of socio-economic impact records as categorised by: **A** alien species causing impacts, **B** broad geographic location of impact, and **C** mechanism of impact; and the number and mechanism of socio-economic impact records as categorised by: **D** alien species causing impacts, and **E** broad geographic location of impact. Alien leporid species: *AR* Arctic hare, *BL* Black-tailed jackrabbit, *CA* Cape hare, *CO* Corsican hare, *EA* Eastern cottontail, *EH* European hare, *ER* European rabbit, *IB* Iberian hare, *IH* Indian hare, *MO* Mountain hare, *SN* Snowshoe hare, *WH* White-tailed jackrabbit. Locations: *OC* Oceania, *EU* Europe, *IS* Island, *SO* South America, *NO* North America. Impact severity categories: *weak* impacts categorised as MC or MN under SEICAT, *moderate* impacts categorised as MO, *severe* impacts categorised as MR or MV. SEICAT impact mechanisms: *AG* agriculture/forestry/horticulture industry, *HE* human health and safety, *MA* material assets, *RE* recreation, *TO* tourism industry

No impact data were found for six alien leporid species, which were classified as DD under SEICAT (Fig. 5A). Most impacts (88%) were ‘weak’, 5% were ‘moderate’ and 7% were ‘severe’. The only species to cause ‘severe’ impacts was the European rabbit.

Contingency table tests did not reveal any significant results (Table 3, tests #13–17). Most impacts were recorded in Oceania (43%), including the only ‘severe’ impacts, and the least were recorded on islands (10%), including the only ‘moderate’ impacts (Fig. 5B). Most socio-economic impacts (76%) affected agriculture (Fig. 5C). The European rabbit was the only species to cause impacts through all five impact mechanisms (Fig. 5D).

## Discussion

### Impact data availability

This is the first study to categorise the severity and type of environmental and socio-economic impacts caused by all known alien leporid species. Relatively few socio-economic impact records were identified, and this may be because SEICAT requires data on how the socio-economic impacts of alien species affect human well-being, and many reports describing socio-economic impacts do not include such information. Indeed, this may be why many alien bird species are categorised as DD under SEICAT (Evans et al. 2020). Another reason may be because some alien leporid species are present in non-English speaking regions, and as our searches were carried out in English, we may have missed reports describing socio-economic impacts in other languages. However, we did not find any socio-economic impact data for several alien leporid species present in English speaking regions. Another possible reason is that invasion scientists are, due to their formal training, more concerned with identifying environmental impacts than socio-economic impacts. If this is true, then the impacts of alien species on human well-being remains a neglected research topic. However, the scarcity of socio-economic impact reports could also be because alien species research tends to focus on those species with the most damaging impacts (Pyšek et al. 2008), and the environmental impacts of alien leporids are more damaging than their socio-economic impacts. This might suggest that research should prioritise environmental impacts over socio-economic impacts. However, environmental and socio-economic impacts are connected, as nature provides important contributions to people and their well-being (Díaz et al. 2018). Thus, the results of this study indicate that the consequences to human well-being arising from environmental impacts are understudied. For example, there are no reports describing how native species extinctions caused by alien leporids affect people.

Several alien leporid species were DD (they had no reported environmental or socio-economic impacts). Impact data are also unavailable for alien species from other taxonomic groups, including amphibians (Measey et al. 2016; Bacher et al. 2018), gastropods (Kesner and Kumschick 2018) and birds (Evans et al. 2018b, 2020). There are several reasons why some alien leporid species are DD. First, they may occupy less-developed regions with limited capacity to undertake research, or remote or inhospitable regions where impacts are difficult to study. This is likely to be why some alien bird species have no reported impacts (Evans et al. 2018b, 2020; Evans and Blackburn 2020). The small number of alien leporid species (12) prevented an analysis of factors that may influence impact data availability, as has been undertaken using a dataset of 415 alien bird species (Evans et al. 2018b). However, the four alien leporid species with no reported environmental impacts are present on mainland USA, Sardinia (Italy) and the Newfoundland Islands (Canada). Three of these species also have no reported socio-economic impacts. The three other species with no reported socio-economic impacts are present on mainland France, Corsica (France), Saint Pierre and Miquelon (French Overseas Territory) and Kent Island (Canada). These are comparatively wealthy, developed and accessible regions of the world, which suggests that human development and geographic isolation may not be a key influence on the availability of impact data.

Second, these DD species may have weak impacts that do not attract research. This is likely to be the case for many alien bird species (Evans et al. 2018b). Consistent with this hypothesis, the reported socio-economic impacts of alien leporids tend to be ‘weak’, with only a small number being ‘moderate’ or ‘severe’. However, their reported environmental impacts tend to be ‘moderate’, which does not support this hypothesis. The vast majority of these ‘moderate’ impacts (and all ‘severe’ impacts) are directly or indirectly caused by grazing. Grazing is an activity common to all alien leporid species (both species with reported impacts and those that are DD). Thus, DD alien leporid species may be causing damaging environmental impacts that are going unnoticed.

Third, DD species may have small alien ranges, which limits their opportunities to cause impacts, and may also reduce the chances that their impacts are noticed and reported. Indeed, DD alien birds tend to have smaller alien ranges than those with reported impacts (Evans et al. 2018b). The European rabbit and European hare have much larger alien ranges than all other alien leporid species, having been introduced to many more locations. Together they caused 77% of all environmental impacts and 86% of all socio-economic impacts. This is likely to be why the number of environmental and socio-economic impacts caused by alien leporid species is positively correlated. As both the environmental and socio-economic impacts of the European rabbit and European hare have been recorded in less-developed regions of the world (e.g. countries in South America, Mauritius, Kiribati), and their reported socio-economic impacts tend to be ‘weak’, it may be that opportunity for impact (associated with alien range size and the number of locations a species is introduced to) has more of an influence on the availability of impact data than human development or impact severity (although it is likely that data availability is to some extent influenced by all three of the above factors). This suggests that DD alien leporid species that have small alien ranges or are restricted to a small number of locations as aliens, may have damaging impacts that are going unnoticed.

### Environmental impacts

Most impacts, and all ‘severe’ impacts, were recorded on islands or mainland Australia or New Zealand. Island ecosystems are vulnerable to the impacts of alien species (Spatz et al. 2017), and alien species are a key driver of species extinctions in Australasia (e.g. Woinarski et al. 2015; Kearney et al. 2018). As a consequence, much effort has been dedicated to identifying and managing their impacts (e.g. https://www.islandconservation.org and https://predatorfreenz.org) which may explain the prevalence for impact records in these regions.

Most ‘moderate’ and ‘severe’ environmental impacts are the consequence of grazing by the European rabbit, which directly affects plants, and indirectly affects animals that occupy habitats associated with these plants. Direct impacts include the extinction of the Waiautoa forget-me-not (*Myosotis laingii*) in New Zealand (Norbury 2005). Indirect impacts include grazing of vegetation on Laysan Island (Northwestern Hawaiian Islands) which caused the extinction of the Laysan millerbird (*Acrocephalus familiaris familiaris*) (BirdLife International 2021). Many more grazing impacts were identified in comparison to indirect impacts, which suggests that indirect impacts caused by grazing are underreported. Indeed, several alien leporid species had reported grazing impacts but no reported indirect impacts. This may be viewed with concern, as reported indirect impacts tended to be more damaging (particularly for birds) than impacts caused by other mechanisms. For example, grazing by the Snowshoe hare on Saint Pierre and Miquelon is a serious threat to biodiversity (Peterson et al. 2005), but there are no reports describing indirect impacts on these islands. For other regions, some indirect grazing impacts were identified which affected a range of species including insects, birds, centipedes, mammals and reptiles. For example, grazing of Silver tussock (*Poa cita*) on New Zealand’s South Island caused a population decline of the critically endangered (CR) Cromwell Chafer beetle (*Prodontria lewisi*) (Norbury 2005).

Some indirect impacts did not result from grazing. For example, Cromwell Chafer beetles are preyed upon by alien Redback spiders (*Latrodectus hasselti*) which build webs in burrows created by European rabbits (Spencer et al. 2017). Other indirect impacts have been attributed to changes in predator–prey dynamics, whereby populations of predatory native species increase due to the availability of alien leporid species as prey, which increases predation pressure on native prey species (the hyperpredation hypothesis) (Courchamp et al. 2000). On Macquarie Island (Australia), this is believed to have resulted in the extinction of the Macquarie rail (*Gallirallus philippensis macquariensis*) (Taylor 1979). Only a small number of examples of this type of impact were identified. Indeed, impacts associated with apparent competition have received relatively little attention in conservation biology (Courchamp et al. 2000). There are likely to be other islands where alien leporid species are causing elevated predation pressure on native prey species. Consumption of alien leporids by alien feral cats (*F. catus*) is positively correlated with latitude, as European rabbits are present on many sub-Antarctic islands (Bonnaud et al. 2011).

Alien leporid species also cause ‘moderate’ environmental impacts through two other mechanisms (competition and hybridisation), although fewer impact records were identified for these mechanisms. This may be because competition and hybridisation tend to involve interactions between taxonomically similar native and alien species, and therefore occur less frequently. Most competition impacts and all hybridisation impacts affected native mammals. Competition tended to be for food, with affected species including the Gray brocket (*Mazama gouazoubira*) in Argentina (Kufner et al. 2008). The European hare has been recorded hybridising with the Irish hare (*L. timidus hibernicus*) in Ireland (Reid and Montgomery 2007), and the Mountain hare (*L. timidus*) in Sweden (Thulin and Tegelström 2002) and Finland (Levänen et al. 2018). Few reports described disease transmission impacts, perhaps because such impacts occur infrequently, and because determining the cause of disease impacts can be difficult. Indeed, few disease impacts have been attributed to alien birds (Evans et al. 2016), prompting suggestions that they are being overlooked (Tompkins and Jakob-Hoff 2011).

### Socio-economic impacts

By far the most frequently reported socio-economic impacts were associated with grazing, which mainly affected agricultural and horticultural crops. These impacts were reported across all five broad locations occupied by alien leporid species. The only three ‘severe’ impact records were for agricultural impacts in Australia and New Zealand, where grazing by the European rabbit caused the permanent abandonment of farms in the first half of the twentieth century (Peden 2008; Buseth and Saunders 2014; CSIRO 2021). All remaining impact records associated with grazing were ‘weak’, as there was no evidence to indicate that they resulted in fewer people participating in agricultural activities (a SEICAT criteria for ‘moderate’ impacts). Nevertheless, some of these impacts have significant financial implications, such as the aforementioned impacts to Australia’s wool industry (Vere et al. 2004). Other minor grazing impacts related to damage to gardens (e.g. Victoria News 2019). Thus, the only severe socio-economic impact records associated with alien leporid species are historical, which may suggest that we have learned to live with and manage the most damaging socio-economic impacts of alien leporids (see also Jernelöv 2017). Nevertheless, the severity of impacts caused by alien species can vary over time (Strayer et al. 2006). It may be influenced, for example, by changes in approaches to their management (Ruscoe et al. 2011) and the arrival of a new alien species which creates conditions that enable the existing alien species to thrive and cause impacts (Spencer et al. 2017). Therefore, continued monitoring of alien species is important to maintain and improve our understanding of their impacts (Jernelöv 2017; Pergl et al. 2020).

The only two ‘moderate’ socio-economic impacts affected recreation and tourism. Aside from the aforementioned impacts on Macquarie Island (Australia), hunting of introduced Mountain hares on the Faroe Islands compromises the safety of hikers and restricts their access to hiking routes (Local.fo 2018). Although these impacts were recorded on islands, fewer socio-economic impacts were identified on islands than would have been expected when compared to environmental impacts. This may be because there are few human populations on some islands occupied by alien leporids (some are uninhabited).

Impacts to health and safety and material assets were ‘weak’; they tended to be associated with the risk of traffic accidents, and damage to cars caused by these accidents, respectively (e.g. The Age 2016). The European rabbit caused impacts through all five impact mechanisms identified; its broad distribution perhaps increasing its opportunity to cause different types of impacts.

## Conclusions

This study is the first to quantify and categorise the environmental and socio-economic impacts caused by alien leporid species using EICAT and SEICAT. Their environmental impacts tend to be damaging, causing declining populations of native species (and species extirpations and extinctions). Native species from a range of taxonomic groups are vulnerable to these impacts. The reported indirect environmental impacts of alien leporid species are particularly damaging and tend to be underreported. This study may inform future research to identify and manage these impacts.

The recorded socio-economic impacts of alien leporid species tended to be less damaging than their environmental impacts. As invasion science research tends to focus on the most damaging impacts caused by alien species, this may be why less data were found for socio-economic impacts. However, it is also likely to be because SEICAT requires data on the way in which alien species affect human well-being, as measured by changes to human activities, and many socio-economic impact reports do not provide this information.

There are many regions of the world where data on the impacts of alien leporid species are unavailable. Furthermore, no environmental impact data were found for one-third of these species, and no socio-economic impact data for half of them. The results of this study suggest that, particularly for environmental impacts, these species may have damaging impacts that are going unnoticed. Although the impacts of alien species are context dependent (Kumschick et al. 2015), it is plausible that the broad impact patterns identified in this study are likely to persist if more impact data were available. This is because alien leporid species (both those with impact data and those without) possess the same mechanisms of impact. This study may therefore help to identify vulnerable native species and human populations in regions where impact data are unavailable. It may also help to predict the consequences of new introductions of alien leproid species within a region.

Our conclusions lead to three broad recommendations for future research on the impacts of alien leporids. First, to build a more complete picture of these impacts, research should identify impacts in regions occupied by alien leporids where impact data are unavailable, and impacts caused by alien leporid species for which no impact data were identified (DD species). Second, measures should be put in place to mitigate for damaging indirect environmental impacts caused by alien leporid species. Third, future research on socio-economic impacts should describe how alien leporid species affect human well-being, as measured by the ways in which they change human activities.

## Supplementary Information

Below is the link to the electronic supplementary material.

Supplementary file1 (PDF 676 kb)
