# Supplementary material for: An assessment of the environmental and socio-economic impacts of alien rabbits and hares
Source: Ambio. 2021 Oct 28;51(5):1314–29. doi: 10.1007/s13280-021-01642-7 (PMC8931149; doi:10.1007/s13280-021-01642-7)
Supplement: Supplementary file 1 — Supplementary file1 (PDF 676 kb) [file 13280_2021_1642_MOESM1_ESM.pdf]

***Ambio***

Supplementary Information
*This supplementary information has not been peer reviewed*

Title: **An assessment of the environmental and socio-economic impacts of alien rabbits and hares**

**Appendix S1: Literature review protocol**

Following Evans et al. (2016), we carried out an online search using search terms within a search string, in conjunction with the specific alien species' scientific and common name(s). For example, the search string for the Snowshoe hare was: ("introduced species", "invasive species", "invasive alien species", "IAS", "alien", "non-native", "non-indigenous", "pest", "feral" and "exotic") AND ("snowshoe hare" OR "snowshoe rabbit" OR "varying hare" OR "*Lepus americanus*"). We used the same search terms and the following scientific and common names for the other alien species:

- Arctic hare, *Lepus arcticus*
- Black-tailed jackrabbit, jackass hare, narrow-gage mule, *Lepus californicus*
- Cape hare, Arabian hare, desert hare, Sardinian hare, *Lepus capensis*
- Corsican hare, Apennine hare, Italian hare, *Lepus corsicanus*
- Eastern cottontail, Florida cottontail, *Sylvilagus floridanus*
- European hare, brown hare, *Lepus europaeus*
- European rabbit, rabbit, *Oryctolagus cuniculus*
- Iberian hare, Granada hare, *Lepus granatensis*
- Indian hare, black-naped hare, *Lepus nigricollis*
- Mountain hare, alpine hare, blue hare, Irish hare, snow hare, white hare, *Lepus timidus*
- White-tailed jackrabbit, prairie hare, Townsend hare, white jack, *Lepus townsendii*

Databases searched:

- Web of Science (http://apps.webofknowledge.com)
- Google (https://www.google.co.uk)
- Google Scholar (https://scholar.google.co.uk)

Other online resources searched:

- IUCN Red List of Threatened Species (http://www.iucnredlist.org)
- CABI Invasive Species Compendium (http://www.cabi.org/isc)
- Global Invasive Species Database (GISD) of the Invasive Species Specialist Group (ISSG) (http://www.issg.org/database/welcome)

We searched for additional references listed in any articles and data sources found, repeating this process to a point where no new sources of data were identified.

**References**

Evans T, Kumschick S, Blackburn TM. 2016. Application of the Environmental Impact Classification for Alien Taxa (EICAT) to a global assessment of alien bird impacts. *Diversity and Distributions* **22**:919–931.

## Appendix S2: Contingency table test results

### *Environmental vs socio-economic impacts*

Table S1: Contingency table (chi-squared test) showing the actual and expected number of environmental and socio-economic impact records for each alien leporid species. Expected values are displayed in italics. Individual $\chi^2$ values are displayed in (parentheses). Due to small sample sizes, impacts for two alien leporid species (the Black-tailed jackrabbit and Iberian hare) were combined to produce the 'Other species' group.

| | **Corsican hare** | **Eastern cottontail** | **European hare** | **European rabbit** | **Indian hare** | **Mountain hare** | **Snowshoe hare** | **Other species** | **Total** |
|---|---|---|---|---|---|---|---|---|---|
| **Environmental impact records** | 9<br>*7.04*<br>(0.54) | 2<br>*3.91*<br>(0.93) | 12<br>*14.87*<br>(0.55) | 105<br>*104.84*<br>(< 0.001) | 7<br>*6.26*<br>(0.09) | 6<br>*5.48*<br>(0.05) | 9<br>*7.04*<br>(0.54) | 1<br>*1.56*<br>(0.2) | 151 |
| **Socio-economic impact records** | 0<br>*1.96*<br>(1.96) | 3<br>*1.09*<br>(3.36) | 7<br>*4.13*<br>(1.99) | 29<br>*29.16*<br>(< 0.001) | 1<br>*1.74*<br>(0.32) | 1<br>*1.52*<br>(0.18) | 0<br>*1.96*<br>(1.96) | 1<br>*0.44*<br>(0.73) | 42 |
| **Total** | 9 | 5 | 19 | 134 | 8 | 7 | 9 | 2 | 193 |

$\chi^2 = 5.04$, degrees of freedom = 7, $P < 0.001$, estimate = 0.08

Table S2: Contingency table (chi-squared test) showing the actual and expected number of ‘weak’, ‘moderate’ and ‘severe’ environmental and socio-economic impact records. Expected values are displayed in italics. Individual $\chi^2$ values are displayed in (parentheses).

| | Environmental impact records | Socio-economic impact records | Total |
|---|---|---|---|
| **Number of ‘weak’ impact records** | 38<br>*58.69*<br>(7.29) | 37<br>*16.32*<br>(26.2) | 75 |
| **Number of ‘moderate’ impact records** | 99<br>*79.02*<br>(5.05) | 2<br>*21.98*<br>(18.16) | 101 |
| **Number of ‘severe’ impact records** | 14<br>*13.3*<br>(0.04) | 3<br>*3.7*<br>(0.13) | 17 |
| **Total** | 151 | 42 | 193 |

$\chi^2$ = 38.73, degrees of freedom = 2, $P < 0.001$, estimate = 0.44

Table S3: Contingency table (chi-squared test) showing the actual and expected number of environmental and socio-economic impact records as distributed by broad geographic location. Expected values are displayed in italics. Individual $\chi^2$ values are displayed in (parentheses).

| | Oceania | Europe | Islands | North America | South America | Total |
|---|---|---|---|---|---|---|
| **Environmental impact records** | 50<br>*53.2*<br>(0.19) | 7<br>*10.95*<br>(1.43) | 88<br>*71.98*<br>(3.57) | 1<br>*5.48*<br>(3.66) | 5<br>*9.39*<br>(2.05) | 151 |
| **Socio-economic impact records** | 18<br>*14.8*<br>(0.69) | 7<br>*3.05*<br>(5.13) | 4<br>*20.02*<br>(12.82) | 6<br>*1.52*<br>(13.16) | 7<br>*2.61*<br>(7.38) | 42 |
| **Total** | 68 | 14 | 92 | 7 | 12 | 193 |

$\chi^2$ = 48.97, degrees of freedom = 4, $P < 0.001$, estimate = 0.32

## *Environmental impacts*

Table S4: Contingency table (chi-squared test) showing the actual and expected number of ‘weak’, ‘moderate’ and ‘severe’ environmental impact records as distributed by alien leporid species. Expected values are displayed in italics. Individual $\chi^2$ values are displayed in (parentheses). Due to small sample sizes, impacts for two alien leporid species (the Eastern cottontail and Iberian hare) were combined to produce the ‘Other species’ group.

| | Corsican hare | European hare | European rabbit | Indian hare | Mountain hare | Snowshoe hare | Other species | Total |
|---|---|---|---|---|---|---|---|---|
| **Number of ‘weak’ impact records** | 9<br>*2.26*<br>(20.03) | 6<br>*3.02*<br>(2.94) | 9<br>*26.42*<br>(11.49) | 7<br>1.76<br>(15.58) | 6<br>1.51<br>(13.35) | 0<br>*2.26*<br>(2.26) | 1<br>*0.75*<br>(0.08) | 38 |
| **Number of ‘moderate’ impact records** | 0<br>*5.9*<br>(5.9) | 6<br>*7.87*<br>(0.44) | 82<br>*68.84*<br>(2.52) | 0<br>*4.59*<br>(4.59) | 0<br>*3.93*<br>(3.93) | 9<br>*5.9*<br>(1.63) | 2<br>*1.97*<br>(< 0.001) | 99 |
| **Number of ‘severe’ impact records** | 0<br>*0.83*<br>(0.83) | 0<br>*1.11*<br>(1.11) | 14<br>*9.74*<br>(1.87) | 0<br>0.65<br>(0.65) | 0<br>0.56<br>(0.56) | 0<br>*0.83*<br>(0.83) | 0<br>*0.28*<br>(0.28) | 14 |
| **Total** | 9 | 12 | 105 | 7 | 6 | 9 | 3 | 151 |

$\chi^2$ = 104.67, degrees of freedom = 12, $P < 0.001$, estimate = 0.39

Table S5: Contingency table (chi-squared test) showing the actual and expected number of ‘weak’, ‘moderate’ and ‘severe’ environmental impact records as distributed by family of affected native plants. Expected values are displayed in italics. Individual $\chi^2$ values are displayed in (parentheses). Due to small sample sizes, impact records associated with 35 plant families were grouped together to form the ‘Other plant families’ category for this test. Impact records for species from unidentified plant families were excluded for this test.

| | **Grass (*Poaceae*) family** | **Other plant families** | **Total** |
|---|---|---|---|
| **Number of ‘weak’ impact records** | 12<br>*8.04*<br>(1.95) | 18<br>*21.96*<br>(0.71) | 30 |
| **Number of ‘moderate’ impact records** | 14<br>*16.62*<br>(0.41) | 48<br>*45.38*<br>(0.15) | 62 |
| **Number of ‘severe’ impact records** | 0<br>*1.34*<br>(1.34) | 5<br>*3.67*<br>(0.49) | 5 |
| **Total** | 26 | 71 | 97 |

$\chi^2$ = 3.97, degrees of freedom = 2, *P* = 0.063, estimate = 0.2

Table S6: Contingency table (chi-squared test) showing the actual and expected number of ‘weak’, ‘moderate’ and ‘severe’ environmental impact records as distributed by class of affected native animals. Expected values are displayed in italics. Individual $\chi^2$ values are displayed in (parentheses). Due to small sample sizes, impacts for three classes of native animal (chilopods, insects and reptiles) were combined to produce the ‘Other classes’ group.

| | **Birds** | **Mammals** | **Other classes** | **Total** |
|---|---|---|---|---|
| **Number of ‘weak’ impact records** | 2<br>*3.5*<br>(0.64) | 4<br>*2.69*<br>(0.64) | 1<br>*0.81*<br>(0.05) | 7 |
| **Number of ‘moderate’ impact records** | 16<br>*18*<br>(0.22) | 15<br>*13.85*<br>(0.1) | 5<br>*4.15*<br>(0.17) | 36 |
| **Number of ‘severe’ impact records** | 8<br>*4.5*<br>(2.72) | 1<br>*3.46*<br>(1.75) | 0<br>*1.04*<br>(1.04) | 9 |
| **Total** | 26 | 20 | 6 | 52 |

$\chi^2$ = 8.68, degrees of freedom = 4, *P* = 0.059, estimate = 0.29

Table S7: Contingency table (chi-squared test) showing the actual and expected number of ‘weak’, ‘moderate’ and ‘severe’ environmental impact records as distributed by animal and plant kingdom. Expected values are displayed in italics. Individual $\chi^2$ values are displayed in (parentheses).

| | **Animal kingdom** | **Plant kingdom** | **Total** |
|---|---|---|---|
| **Number of ‘weak’ impact records** | 7<br>*13.09*<br>(2.83) | 31<br>*24.91*<br>(1.49) | 38 |
| **Number of ‘moderate’ impact records** | 36<br>*34.09*<br>(0.11) | 63<br>*64.91*<br>(0.06) | 99 |
| **Number of ‘severe’ impact records** | 9<br>*4.82*<br>(3.62) | 5<br>*9.18*<br>(1.9) | 14 |
| **Total** | 52 | 99 | 151 |

$\chi^2$ = 9.04, degrees of freedom = 2, *P* = 0.007, estimate = 0.24

Table S8: Contingency table (chi-squared test) showing the actual and expected number of 'weak', 'moderate' and 'severe' environmental impact records as distributed by broad geographic location. Expected values are displayed in italics. Individual $\chi^2$ values are displayed in (parentheses). Due to small sample sizes, impacts for North and South America were combined to produce the 'Americas' group.

| | **Oceania** | **Europe** | **Islands** | **Americas** | **Total** |
|---|---|---|---|---|---|
| **Number of 'weak' impact records** | 8<br>*12.58*<br>(1.67) | 1<br>*1.76*<br>(0.33) | 27<br>*22.15*<br>(1.06) | 2<br>*1.51*<br>(0.16) | 38 |
| **Number of 'moderate' impact records** | 38<br>*32.78*<br>(0.83) | 6<br>*4.59*<br>(0.43) | 51<br>*57.7*<br>(0.78) | 4<br>*3.93*<br>(0.001) | 99 |
| **Number of 'severe' impact records** | 4<br>*4.64*<br>(0.09) | 0<br>*0.65*<br>(0.65) | 10<br>*8.16*<br>(0.42) | 0<br>*0.56*<br>(0.56) | 14 |
| **Total** | 50 | 7 | 88 | 6 | 151 |

$\chi^2 = 9.06$, degrees of freedom = 6, $P = 0.096$, estimate = 0.15

Table S9: Contingency table (chi-squared test) showing the actual and expected number of 'weak', 'moderate' and 'severe' environmental impact records as distributed by EICAT impact mechanism. Expected values are displayed in italics. Individual $\chi^2$ values are displayed in (parentheses).

| | **Competition** | **Grazing / herbivory / browsing** | **Indirect** | **Other mechanisms** | **Total** |
|---|---|---|---|---|---|
| **Number of 'weak' impact records** | 3<br>*2.26*<br>(0.24) | 31<br>*24.16*<br>(1.94) | 3<br>*10.57*<br>(5.42) | 1<br>*1.01*<br>(< 0.001) | 38 |
| **Number of 'moderate' impact records** | 5<br>*5.9*<br>(0.14) | 60<br>*62.94*<br>(0.14) | 31<br>*27.54*<br>(0.44) | 3<br>*2.62*<br>(0.05) | 99 |
| **Number of 'severe' impact records** | 1<br>*0.83*<br>(0.03) | 5<br>*8.9*<br>(1.71) | 8<br>*3.89*<br>(4.33) | 0<br>*0.37*<br>(0.37) | 14 |
| **Total** | 9 | 96 | 42 | 4 | 151 |

$\chi^2 = 21.1$, degrees of freedom = 6, $P = 0.002$, estimate = 0.23

Table S10: Contingency table (chi-squared test) showing the actual and expected number of environmental impact records for each alien leporid species as distributed by EICAT impact mechanism. Expected values are displayed in italics. Individual $\chi^2$ values are displayed in (parentheses). Due to small sample sizes, impacts for two impact mechanisms (transmission of diseases and hybridisation) were combined to produce the 'Other mechanisms' group, and impacts for two alien leporid species (the Eastern cottontail and Iberian hare) were combined to produce the 'Other species' group.

| | **Corsican hare** | **European hare** | **European rabbit** | **Indian hare** | **Mountain hare** | **Snowshoe hare** | **Other species** | **Total** |
|---|---|---|---|---|---|---|---|---|
| **Competition** | 0 | 4 | 4 | 0 | 0 | 0 | 1 | 9 |
| | *0.54* | *0.72* | *6.26* | *0.42* | *0.36* | *0.54* | *0.18* | |
| | (0.54) | (15.09) | (0.81) | (0.42) | (0.36) | (0.54) | (3.77) | |
| **Grazing** | 9 | 5 | 60 | 7 | 6 | 9 | 0 | 96 |
| | *5.72* | *7.63* | *66.76* | *4.45* | *3.81* | *5.72* | *1.91* | |
| | (1.88) | (0.91) | (0.68) | (1.46) | (1.25) | (1.88) | (1.91) | |
| **Indirect** | 0 | 0 | 41 | 0 | 0 | 0 | 1 | 42 |
| | *2.5* | *3.34* | *29.21* | *1.95* | *1.67* | *2.5* | *0.83* | |
| | (2.5) | (3.34) | (4.76) | (1.95) | (1.67) | (2.5) | (0.03) | |
| **Other mechanisms** | 0 | 3 | 0 | 0 | 0 | 0 | 1 | 4 |
| | *0.24* | *0.32* | *2.78* | *0.19* | *0.16* | *0.24* | *0.08* | |
| | (0.24) | (22.63) | (2.78) | (0.19) | (0.16) | (0.24) | (10.66) | |
| **Total** | 9 | 12 | 105 | 7 | 6 | 9 | 3 | 151 |

$\chi^2$ = 76.2, degrees of freedom = 18, $P < 0.001$, estimate = 0.31

Table S11: Contingency table (chi-squared test) showing the actual and expected number of environmental impact records for each native animal class as distributed by EICAT impact mechanism. Expected values are displayed in italics. Individual $\chi^2$ values are displayed in (parentheses). Due to small sample sizes, impacts for two impact mechanisms (transmission of diseases and hybridisation) were combined to produce the 'Other mechanisms' group, and impacts for three classes of native animal (chilopods, insects and reptiles) were combined to produce the 'Other classes' group.

| | **Birds** | **Mammals** | **Other classes** | **Total** |
|---|---|---|---|---|
| **Competition** | 1 | 8 | 0 | 9 |
| | *4.5* | *3.46* | *1.04* | |
| | (2.72) | (5.95) | (1.04) | |
| **Indirect** | 25 | 8 | 6 | 39 |
| | *19.5* | *15* | *4.5* | |
| | (1.55) | (3.27) | (0.5) | |
| **Other mechanisms** | 0 | 4 | 0 | 4 |
| | *2* | *1.54* | *0.46* | |
| | (2) | (3.94) | (0.46) | |
| **Total** | 26 | 20 | 6 | 52 |

$\chi^2$ = 25.28, degrees of freedom = 4, $P < 0.001$, estimate = 0.49

Table S12: Contingency table (chi-squared test) showing the actual and expected number of environmental impact records associated with each EICAT impact mechanism as distributed by broad geographic location. Expected values are displayed in italics. Individual $\chi^2$ values are displayed in (parentheses). Due to small sample sizes, impacts for North and South America were combined to produce the 'Americas' group, and impacts for two impact mechanisms (transmission of diseases and hybridisation) were combined to produce the 'Other mechanisms' group.

| | **Oceania** | **Europe** | **Islands** | **Americas** | **Total** |
|---|---|---|---|---|---|
| **Competition** | 3<br>*2.98*<br>(< 0.001) | 2<br>*0.42*<br>(6) | 1<br>*5.25*<br>(3.44) | 3<br>*0.36*<br>(19.52) | 9 |
| **Grazing** | 30<br>*31.79*<br>(0.1) | 0<br>*4.45*<br>(4.45) | 63<br>*55.95*<br>(0.89) | 3<br>*3.81*<br>(0.17) | 96 |
| **Indirect** | 17<br>*13.91*<br>(0.69) | 1<br>*1.95*<br>(0.46) | 24<br>*24.48*<br>(0.009) | 0<br>*1.67*<br>(1.67) | 42 |
| **Other mechanisms** | 0<br>*1.32*<br>(1.32) | 4<br>*0.19*<br>(78.47) | 0<br>*2.33*<br>(2.33) | 0<br>*0.16*<br>(0.16) | 4 |
| **Total** | 50 | 7 | 88 | 6 | 151 |

$\chi^2$ = 38.33, degrees of freedom = 9, $P < 0.001$, estimate = 0.29

## *Socio-economic impacts*

Table S13: Contingency table (chi-squared test) showing the actual and expected number of 'weak' and 'moderate' or 'severe' socio-economic impact records as distributed by alien leporid species. Expected values are displayed in italics. Individual $\chi^2$ values are displayed in (parentheses). Due to small sample sizes, impacts for three alien leporid species (the Black-tailed jackrabbit, Indian hare and Mountain hare) were combined to produce the 'Other species' group, and 'moderate' and 'severe' impact records were combined.

| | **Eastern cottontail** | **European hare** | **European rabbit** | **Other species** | **Total** |
|---|---|---|---|---|---|
| **Number of 'weak' impact records** | 3<br>*2.64*<br>(0.05) | 7<br>*6.17*<br>(0.11) | 25<br>*25.55*<br>(0.01) | 2<br>*2.64*<br>(0.16) | 37 |
| **Number of 'moderate' or 'severe' impact records** | 0<br>*0.36*<br>(0.36) | 0<br>*0.83*<br>(0.83) | 4<br>*3.45*<br>(0.09) | 1<br>*0.36*<br>(1.16) | 5 |
| **Total** | 3 | 7 | 23 | 3 | 42 |

$\chi^2$ = 1.39, degrees of freedom = 3, $P$ = 0.624, estimate = 0.13

Table S14: Contingency table (chi-squared test) showing the actual and expected number of ‘weak’, and ‘moderate’ or ‘severe’ socio-economic impact records as distributed by broad geographic location. Expected values are displayed in italics. Individual $\chi^2$ values are displayed in (parentheses). Due to small sample sizes ‘moderate’ and ‘severe’ impact records were combined.

| | **Oceania** | **Europe** | **Islands** | **North America** | **South America** | **Total** |
|---|---|---|---|---|---|---|
| **Number of ‘weak’ impact records** | 15<br>*15.86*<br>(0.05) | 7<br>*6.17*<br>(0.11) | 2<br>*3.52*<br>(0.66) | 6<br>*5.29*<br>(0.1) | 7<br>*6.17*<br>(0.11) | 37 |
| **Number of ‘moderate’ or ‘severe’ impact records** | 3<br>*2.14*<br>(0.34) | 0<br>*0.83*<br>(0.83) | 2<br>*0.48*<br>(4.88) | 0<br>*0.71*<br>(0.71) | 0<br>*0.83*<br>(0.83) | 5 |
| **Total** | 18 | 7 | 4 | 6 | 7 | 42 |

$\chi^2 = 5.63$, degrees of freedom = 4, $P = 0.176$, estimate = 0.23

Table S15: Contingency table (chi-squared test) showing the actual and expected number of ‘weak’, and ‘moderate’ or ‘severe’ socio-economic impact records as distributed by SEICAT impact mechanism. Expected values are displayed in italics. Individual $\chi^2$ values are displayed in (parentheses). Due to small sample sizes, impact records for four SEICAT impact mechanisms (impacts to health and safety, material assets, the tourism industry and recreation) were combined to form the ‘Other mechanisms’ group, and ‘moderate’ and ‘severe’ impact records were combined.

| | **Agriculture / horticulture / forestry** | **Other mechanisms** | **Total** |
|---|---|---|---|
| **Number of ‘weak’ impact records** | 29<br>*28.19*<br>(0.02) | 8<br>*8.81*<br>(0.07) | 37 |
| **Number of ‘moderate’ or ‘severe’ impact records** | 3<br>*3.81*<br>(0.17) | 2<br>*1.19*<br>(0.55) | 5 |
| **Total** | 32 | 10 | 42 |

$\chi^2 = 0.6$, degrees of freedom = 1, $P = 0.577$, estimate = 0.12

Table S16: Contingency table (chi-squared test) showing the actual and expected number of socio-economic impact records for each alien leporid species as distributed by SEICAT impact mechanism. Expected values are displayed in italics. Individual $\chi^2$ values are displayed in (parentheses). Due to small sample sizes, impacts for impacts for three alien leporid species (the Black-tailed jackrabbit, Indian hare and Mountain hare) were combined to produce the 'Other species' group, and four SEICAT impact mechanisms (impacts to health and safety, material assets, the tourism industry and recreation) were combined to form the 'Other mechanisms' group.

| | **Eastern cottontail** | **European hare** | **European rabbit** | **Other species** | **Total** |
|---|---|---|---|---|---|
| **Agriculture / horticulture / forestry** | 2<br>*2.29*<br>(0.04) | 7<br>*5.33*<br>(0.52) | 22<br>*22.1*<br>(< 0.001) | 1<br>*2.29*<br>(0.72) | 32 |
| **Other mechanisms** | 1<br>*0.71*<br>(0.11) | 0<br>*1.67*<br>(1.67) | 7<br>*6.9*<br>(0.001) | 2<br>*0.71*<br>(2.31) | 10 |
| **Total** | 3 | 7 | 29 | 3 | 42 |

$\chi^2 = 2.37$, degrees of freedom = 3, $P = 0.298$, estimate = 0.17

Table S17: Contingency table (chi-squared test) showing the actual and expected number of socio-economic impact records associated with each SEICAT impact mechanism as distributed by broad geographic location. Expected values are displayed in italics. Individual $\chi^2$ values are displayed in (parentheses). Due to small sample sizes impact records for four SEICAT impact mechanisms (impacts to health and safety, material assets, the tourism industry and recreation) were combined to form the 'Other mechanisms' group.

| | **Oceania** | **Europe** | **Islands** | **North America** | **South America** | **Total** |
|---|---|---|---|---|---|---|
| **Agriculture / horticulture / forestry** | 15<br>*13.71*<br>(0.12) | 5<br>*5.33*<br>(0.02) | 2<br>*3.05*<br>(0.36) | 3<br>*4.57*<br>(0.54) | 7<br>*5.33*<br>(0.52) | 32 |
| **Other mechanisms** | 3<br>*4.29*<br>(0.39) | 2<br>*1.67*<br>(0.07) | 2<br>*0.95*<br>(1.15) | 3<br>*1.43*<br>(1.73) | 0<br>*1.67*<br>(1.67) | 10 |
| **Total** | 18 | 7 | 4 | 6 | 7 | 42 |

$\chi^2 = 5.32$, degrees of freedom = 4, $P = 0.23$, estimate = 0.23

**Appendix S3**

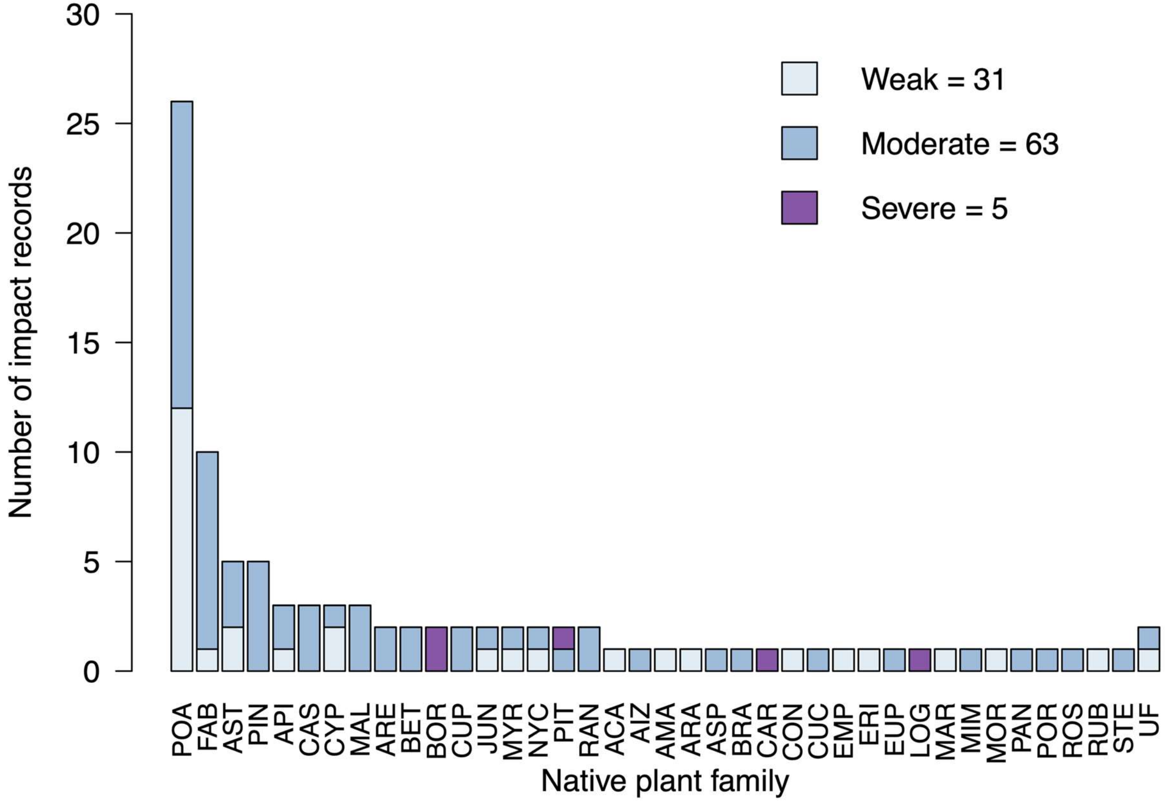


Figure S1: The number and severity of impact records as categorised by native plant family sustaining impacts. Native plant family: ACA = *Acanthaceae*; AIZ = *Aizoaceae*; AMA = *Amaranthaceae*; API = *Apiaceae*; ARA = *Araliaceae*; ARE = *Arecaceae*; ASP = *Asphodelaceae*; AST = *Asteraceae*; BET = *Betulaceae*; BOR = *Boraginaceae*; BRA = *Brassicaceae*; CAR = *Caryophyllaceae*; CAS = *Casuarinaceae*; CON = *Convolvulaceae*; CUC = *Cucurbitaceae*; CUP = *Cupressaceae*; CYP = *Cyperaceae*; EMP = *Empetraceae*; ERI = *Ericaceae*; EUP = *Euphorbiaceae*; FAB = *Fabaceae*; JUN = *Juncaceae*; LOG = *Loganiaceae*; MAL = *Malvaceae*; MAR = *Marattiaceae*; MIM = *Mimosaceae*; MOR = *Moraceae*; MYR = *Myrtaceae*; NYC = *Nyctaginaceae*; PAN = *Pandanaceae*; PIN = *Pinaceae*; PIT = *Pittosporaceae*; POA = *Poaceae*; POR = *Portulacaceae*; RAN = *Ranunculaceae*; ROS = *Rosaceae*; RUB = *Rubiaceae*; STE = *Sterculiaceae*; UF = Unidentified family. Impact severity categories: Weak = impacts categorised as MC or MN under EICAT; Moderate = impacts categorised as MO; Severe = impacts categorised as MR or MV.
